# Association of Plasma Hemoglobin A1c with Improvement of Cognitive Functions by Probiotic *Bifidobacterium breve* Supplementation in Healthy Adults with Mild Cognitive Impairment

**DOI:** 10.3233/JAD-201488

**Published:** 2021-05-18

**Authors:** Francois Bernier, Kazuya Ohno, Noriko Katsumata, Takashi Shimizu, Jinzhong Xiao

**Affiliations:** Morinaga Milk Industry Co., Ltd., Next Generation Science Institute, Kanagawa, Japan

**Keywords:** Bifidobacterium, clinical trial, HbA1c, dementia, memory, mild cognitive impairment, probiotics

## Abstract

We demonstrated the benefit of the probiotic strain, *Bifidobacterium breve* MCC1274 (synonym *B. breve* A1), at improving cognition in our previous double-blind, placebo-controlled clinical study. Analysis of the association of blood parameters changes with the improvement of cognitive function revealed an inverse correlation of HbA1c with total RBANS score amelioration after the study only in the probiotic group (*ρ*= –0.4218, *p* = 0.0067). A stratified analysis based on baseline HbA1c with a median value showed a more remarkable benefit by the probiotic supplementation in the higher median subgroup. These data support the mechanism of anti-inflammation in improving cognition by the probiotic strain.

## INTRODUCTION

The term hemoglobin A1c (HbA1C) refers to glycated hemoglobin. The process occurs when our body’s red blood cells harvest circulating glucose in the bloodstream [1]. It has been demonstrated that the quantity of glucose combined with hemoglobin also reflects the total sugar levels in our system. Since red blood cells survive for up to 8–12 weeks before they are renewed, measuring HbA1c can be used to indicate the average blood glucose over that period. This marker is handy to diagnose people at risk of developing diabetes mellitus, also known as Type 2 diabetes [1]. The usual range of HbA1c (according to the National Glycohemoglobin Standardization Program (NGSP), http://www.ngsp.org) for people without diabetes is < 5.5%, and levels from 5.6%to < 6.4%signals an increased risk of developing diabetes, while levels of 6.5%or higher indicate diabetes and the greater risk of developing diabetes-related complications in Japan [2]. Numerous studies were also able to find an association between higher HbA1c levels and the development of inflammation-related dementia such as Alzheimer’s disease (AD) [3], which is in some circles referred to as Type 3 diabetes [4]. It is well recognized that AD brains have declining uptake of blood glucose as demonstrated in several studies utilizing Fluorodeoxyglucose (FDG)-positron emission tomography (FDG-PET), and this to decline is well associated with a decrease in memory and cognitive performances. There are multiple possible reasons for this: changes in brain insulin receptor levels [5], impairment of blood distribution due to damaged vessels and capillaries [6], and damages to mitochondria due to brain inflammation, affecting brain cells’ survival [7]. While several indications are suggesting the link between dementia, glucose metabolism, and inflammation [8], no precise treatment has been identified that could prevent or reverse the course of those syndromes, including mild cognitive impairment (MCI).

MCI is prevalent in people over 65 years old [9]. It is defined as a decline of cognitive functions associated with the risk of developing sporadic AD or other dementia within a few years if left untreated either by life changes or therapeutic interventions [10].

Probiotics are live microorganisms that have the potential to help to treat several mental illnesses [11]. They are also referred to as friendly or healthy bacteria supplied through foods, beverages, and dietary supplements. One of their reported benefits has been associated with anti-inflammatory effect [12] and correction of gut microbiota dysbiosis [13], that is proposed to be a driver of inflammation and recently shown to correlate with amyloid deposition in the brain of living subjects suffering from AD [14].

We have recently conducted a clinical trial to evaluate the effectiveness of probiotic *Bifidobacterium breve* MCC1274 (synonym *B. breve* A1) in physically healthy subjects with suspected MCI [15]. The trial enrolled subjects with no history of major illnesses such as cancer or cardiovascular diseases but with demonstrated mild cognitive impairment as assessed by the Mini-Mental State Examination (MMSE) and Repeatable Battery for the Assessment of Neuropsychological Status (RBANS) tests, which are well-established methods to detect even slight declines of memory functions. All trial participants were non-obese and fasting glucose levels at the beginning and the end of the study were at a normal range that did not suggest any insulin-resistance in both the placebo and probiotic cohort. The trial successfully met both its primary (RBANS) and secondary endpoints (JMCIS). In that trial, subjects saw a significant improvement in several memory parameters such as immediate memory, delayed memory, and visuospatial/constructional memory without any detrimental side effects as assessed by the physicians who conducted the study. Given the association between HbA1c and the risk of developing dementia as mentioned above, we were interested in a follow-up post-study analysis to see if we could find any correlation of this marker or other safety biomarkers with memory improvement of the study participants that received either *B. breve* MCC1274 daily or a placebo for 16 weeks.

## MATERIAL AND METHODS

### Study design and data analysis

All details of the clinical study design were described previously [15]. Pearson’s *ρ* values and *p*-values were calculated using R software (Ver. 3.6.0). A stratified analysis based on baseline HbA1c with a median value and statistical analysis of the saf-ety biomarkers was performed with Student’s *t*-test. A complete description of the trial and methodolog-ies was published recently in this same journal and is freely available for download at this url: https://content.iospress.com/articles/journal-of-alzheimers-disease/jad200488. Briefly, in this randomized, double-blind, placebo-controlled trial, 80 healthy older adults with MCI were allocated to receive either probiotic (*B. breve* MCC1274, 2×10^10^ CFU) or placebo for 16 weeks. Cognitive functions were assessed by RBANS and the Japanese version of the MCI Screen (JMCIS) tests before and after the study.

## RESULTS

Changes in blood parameters measured throughout the study for safety evaluation are shown in Supplementary Table 1. There were no significant changes from baseline for all markers except for albumin, which tended to be improved during the 16 weeks of treatment in the probiotic group and the changed values from baseline were significantly different between the two groups. We used this information to compare any blood parameters’ change with changes in RBANS total score (Table 1). No significant correlating changes are seen for most markers with RBANS total score amelioration except for an association in the changes of albumin levels in the placebo group and HbA1c in the probiotic group (Table 1).

**Table 1:** Correlation of changes of blood parameters with changes of RBANS total scores

	Probiotics		Placebo	
	*ρ*	*p*	*ρ*	*p*
Triglyceride	–0.1948	0.2285	0.0730	0.6586
Total-cholesterol	0.1355	0.4046	–0.0289	0.8612
Blood urea nitrogen	0.2603	0.1048	0.0074	0.9643
Total-Bil	0.2272	0.1587	0.0672	0.6845
TP	0.0656	0.6875	–0.0470	0.7763
Alb	0.1583	0.3293	–0.4051	0.0105^*^
ALP	–0.0194	0.9057	0.0188	0.9098
LDH	0.1080	0.5073	–0.1245	0.4503
γ-GTP	–0.2773	0.0832	–0.0013	0.9938
AST	–0.1741	0.2828	–0.1153	0.4847
ALT	–0.1674	0.3018	0.0720	0.6630
CREA	0.0741	0.6496	0.0357	0.8293
Uric acid	0.2765	0.0841	–0.1724	0.2940
LDL-cholesterol	–0.1389	0.3926	–0.1342	0.4152
Blood-glucose	–0.2336	0.1468	–0.1033	0.5314
HDL-cholesterol	0.1995	0.2172	0.0902	0.5850
HbA1c	–0.4218	0.0067^**^	0.2205	0.1774

Correlation (*ρ* and *p* values) are by Pearson analysis. ^*^p < 0.05; ^**^p < 0.01.

**Fig. 1:**
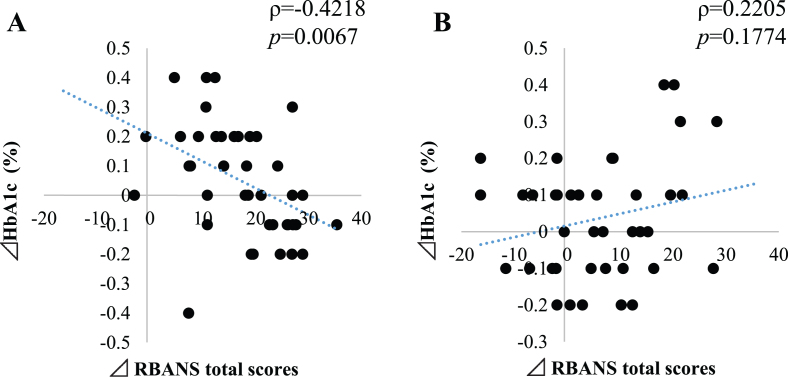
Correlation between changes of RBANS total scores with HbA1c of probiotic group (A) and placebo group (B). Y-axis represents percentage change in HbA1c and the X-axis represents the changes in RBANS total score. *ρ* represents the rho correlation and *p* represents the statistical significance of the correlation (Pearson analysis).

The levels of HbA1c at baseline were within the usual ranges for people without diabetes (4.5–6.0%). The changed values from baseline of HbA1c showed a significant negative correlation (*ρ*= –0.4218, *p* = 0.006) with the changes of RBANS total score, indicating a relationship between an improvement of cognitive functions and a reduction of HbA1c over baseline for the probiotic group but not in the placebo group (Table 1 and Fig. 1A, B). Serum albumin showed a significant negative correlation with RBANS total score changes in the placebo group (*ρ*= –0.4051, *p* = 0.0105) (Table 1).

**Fig. 2:**
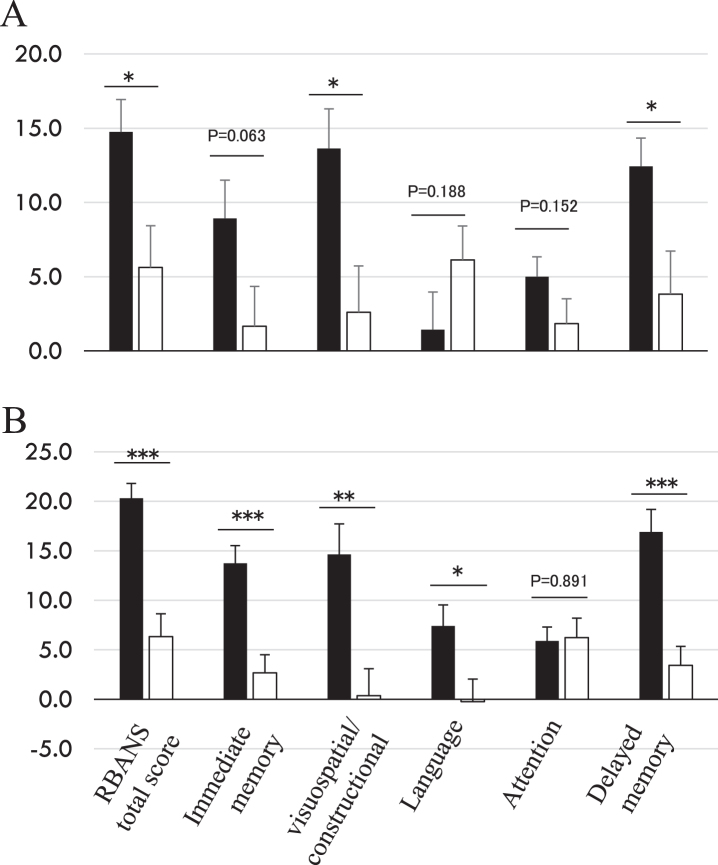
Changes of RBANS scores at 16 weeks from baseline. Stratified analysis based on baseline HbA1c median value (= 5.4%), lower group (A, < 5.4, probiotic *n* = 21, placebo *n* = 18) and higher group (B, ≥5.4, probiotic *n* = 19, placebo *n* = 21). ■: probiotic group; □: placebo group. Y-axis indicates RBANS score change from baseline. ^*^p < 0.05, ^**^p < 0.001, ^***^p < 0.0001, inter-group difference, Student’s *t*-test.

RBANS total score represents a composite score summarized from five domain subscores: immediate memory, visuospatial/constructional, language, attention, and delayed memory. Since higher levels of serum HbA1c is linked to dementia [3], we performed a stratified analysis based on baseline HbA1c with a median value of (= 5.4%) to see if any more specific cognitive parameters improvement were associated with HbA1c changes. All RBANS subscores show a more remarkable significant amelioration in the probiotic group with a higher HbA1c median value greater than 5.4%(Fig. 2A, B) except for attention. Language score was improved only in the higher HbA1c group, something we had not noticed before such stratification in our initial report [15].

## DISCUSSION

The follow-up analysis we conducted reveals that lowering HbA1c serum concentrations correlates with amelioration of cognitive functions as measured with RBANS total score. As reported previously, HbA1C levels in non-diabetic subjects are associated with C-reactive protein concentration [16], an established inflammation marker that shows to be elevated in MCI subjects [17]. While our study did not measure CRP levels, previous preclinical studies with *B. breve* MCC1274 clearly showed that this probiotic has anti-inflammatory properties [18] and hence, we speculate that its positive effect in MCI subjects is linked to a reduction of brain inflammation and reflects the relation with HbA1c changes.

Subtle declines in albumin levels below < 4.05 g/dl have been reported previously [19] to be associated with the risk of developing MCI and AD. The reduction seen in the placebo group that correlates with improvement of memory as measured with RBANS for 16 weeks is difficult to interpret since we observed a placebo effect in the study [15]. Interestingly, the absence of any significant correlation of albumin with RBANS in the probiotic group over baseline (*p* value = 0.3293) perhaps suggests that *B. breve* A1 can slowdown MCI symptoms progression, as reflected by preventing albumin levels declines (*p* = 0.06, Supplementary Table 1).

Although our study enrolled healthy individuals with no signs of diabetes, there is a possibility that participants may have had early signs of insulin resistance as observed by Petersen et al. previously in a similar healthy Caucasian population [20]. We will address this limitation in future studies by conducting a glucose tolerance test that would reveal any early signs of insulin resistance. This assessment could help us understand if B. breve MCC1274 consumption could improve mitochondrial activity linked to impaired brain insulin signaling and inflammation, a deficiency observed in a healthy elderly population and people with dementia previously [21].

## CONCLUSION

We previously reported that 16-week supplementation of the probiotic *B. breve* MCC1274 was safe and effective at improving the participants’ cognitive functions as assesses by RBANS and JMCIS [15]. Further analysis of the safety blood parameters revealed that lowering of HbA1c is associated with improved cognitive functions as measured with RBANS in physically healthy subjects with suspected MCI and suggests the possible mechanism of the effect of probiotics on memory and at reducing inflammation. Further clinical studies using several other markers of inflammation are needed to confirm this conclusion. Our findings also support the hypothesis that HbA1c lowering is a predictor for cognitive impairment amelioration in a non-diabetic healthy population.

## DISCLOSURE STATEMENT

Authors’ disclosures available online (https://www.j-alz.com/manuscript-disclosures/20-1488r2).

## Supplementary Material

Supplementary Material
