# Supplementary material for: Association of Plasma Hemoglobin A1c with Improvement of Cognitive Functions by Probiotic Bifidobacterium breve Supplementation in Healthy Adults with Mild Cognitive Impairment
Source: J Alzheimers Dis. 2021 May 18;81(2):493–7. doi: 10.3233/JAD-201488 (PMC8203240; doi:10.3233/JAD-201488)
Supplement: Supplementary Material [file jad-81-jad201488-s001.pdf]

Supplementary Material

Association of Plasma Hemoglobin A1c with Improvement of Cognitive Functions by Probiotic *Bifidobacterium breve* Supplementation in Healthy Adults with Mild Cognitive Impairment

Supplementary Table 1. Changes of blood parameters during the test period

	Probiotics			Placebo			Changes from baseline		
	Baseline	16 weeks	p	Baseline	16 weeks	p	Probiotics	Placebo	p
Triglyceride (mg/dL)	85.6 (0.6)	89.1 (9.2)	0.98	91.9 (7.5)	89.4 (7.3)	0.6	3.6 (38.4)	-2.7 (30.5)	0.42
Total-Cholesterol (mg/dL)	221.7 (5.9)	230.7 (5.7)	0.15	216.7 (4.9)	219.6 (5.1)	0.52	9.1 (19.3)	3.0 (18.9)	0.16
Blood urea nitrogen (mg/dL)	15.0 (0.6)	15.0 (0.6)	0.29	14.0 (0.6)	14.2 (0.5)	0.19	0 (2.9)	0.3 (3.0)	0.63
Total-Bil (mg/dL)	0.81 (0.05)	0.73 (0.05)	0.34	0.75 (0.04)	0.67 (0.04)	0.42	-0.1 (0.3)	-0.1 (0.2)	0.79
TP (g/dL)	7.27 (0.06)	7.39 (0.07)	0.68	7.23 (0.06)	7.35 (0.06)	0.68	0.1 (0.3)	0.1 (0.3)	0.94
Alb (g/dL)	4.34 (0.04)	4.38 (0.04)	0.06	4.33 (0.03)	4.28 (0.04)	0.96	0 (0.2)	-0.1 (0.2)	0.0232
ALP (units/L)	202.8 (8.3)	214.8 (9.0)	0.77	202.5 (7.7)	211.3 (7.8)	0.98	12.0 (21.2)	6.5 (21.4)	0.26
LDH (units/L)	181.1 (4.8)	188.5 (4.4)	0.95	182.7 (4.1)	188.9 (5.0)	0.8	7.4 (14.9)	5.8 (17.4)	0.65
γ-GTP (units/L)	23.0 (1.9)	24.1 (1.9)	0.21	25.7 (2.2)	28.4 (2.9)	0.35	1.1 (4.8)	2.6 (9.5)	0.38
AST (units/L)	20.7 (1.0)	23.0 (0.9)	0.55	22.3 (0.9)	23.8 (1.0)	0.25	2.3 (4.0)	1.4 (5.6)	0.46
ALT (units/L)	17.5 (1.4)	19.0 (1.2)	0.92	17.7 (1.0)	19.2 (1.3)	0.88	1.6 (6.3)	1.3 (4.9)	0.87
CREA (mg/dL)	0.77 (0.03)	0.77 (0.03)	0.38	0.74 (0.02)	0.74 (0.02)	0.39	0 (0.1)	0 (0.1)	0.87
Uric acid (mg/dL)	5.21 (0.2)	5.09 (0.2)	0.85	5.38 (0.2)	5.14 (0.2)	0.52	-0.1 (0.6)	-0.3 (0.6)	0.27
LDL-Cholesterol (mg/dL)	135.5 (5.8)	133.7 (5.2)	0.49	133.1 (5.6)	128.3 (5.8)	0.76	-1.9 (16.2)	-4.9 (19.4)	0.45
Blood-glucose (mg/dL)	89.0 (1.2)	91.0 (0.9)	0.25	88.7 (1.1)	92.9 (1.4)	0.89	2.1 (5.5)	4.0 (7.0)	0.17
HDL-Cholesterol (mg/dL)	73.5 (2.4)	76.3 (2.7)	0.18	71.8 (2.6)	71.1 (2.7)	0.64	2.9 (8.6)	-0.5 (7.9)	0.08
HbA1c (%)	5.34 (0.05)	5.4 (0.04)	0.56	5.32 (0.05)	5.4 (0.04)	0.72	0 (0.2)	0 (0.2)	0.77

Values are indicated as mean (SD). p values are by Student's t test for inter-group differences.
